# Polluted lake restoration to promote sustainability in the Yangtze River Basin, China

**DOI:** 10.1093/nsr/nwab207

**Published:** 2021-11-24

**Authors:** Boqiang Qin, Yunlin Zhang, Jianming Deng, Guangwei Zhu, Jianguo Liu, David P Hamilton, Hans W Paerl, Justin D Brookes, Tingfeng Wu, Kai Peng, Yizhou Yao, Kan Ding, Xiaoyan Ji

**Affiliations:** State Key Laboratory of Lake Science and Environment, Nanjing Institute of Geography and Limnology, Chinese Academy of Sciences, China; School of Geography and Ocean Science, Nanjing University, China; Nanjing Zhongke Deep Insight Institute Co. Ltd., China; State Key Laboratory of Lake Science and Environment, Nanjing Institute of Geography and Limnology, Chinese Academy of Sciences, China; State Key Laboratory of Lake Science and Environment, Nanjing Institute of Geography and Limnology, Chinese Academy of Sciences, China; State Key Laboratory of Lake Science and Environment, Nanjing Institute of Geography and Limnology, Chinese Academy of Sciences, China; Center for Systems Integration and Sustainability, Department of Fisheries and Wildlife, Michigan State University, USA; Australian Rivers Institute, Griffith University, Australia; Institute of Marine Sciences, University of North Carolina at Chapel Hill, USA; College of the Environment, Hohai University, China; Water Research Centre, School of Biological Science, University of Adelaide, Australia; State Key Laboratory of Lake Science and Environment, Nanjing Institute of Geography and Limnology, Chinese Academy of Sciences, China; State Key Laboratory of Lake Science and Environment, Nanjing Institute of Geography and Limnology, Chinese Academy of Sciences, China; State Key Laboratory of Lake Science and Environment, Nanjing Institute of Geography and Limnology, Chinese Academy of Sciences, China; State Key Laboratory of Lake Science and Environment, Nanjing Institute of Geography and Limnology, Chinese Academy of Sciences, China; China National Environmental Monitoring Centre (CNEMC), China

## Abstract

China has made a concerted effort to successfully improve water quality of rivers, but lake water quality has not improved. Lakes require controls on both catchment external nutrient loads and in-lake internal loads, where nature-based solutions are coupled with engineered systems to achieve the United Nations Sustainable Development Goals (SDGs).

China has sought to address water pollution in the last decade by introducing a wide range of laws and regulations (Table S1), which led to nationwide water quality improvement [1]. However, recent quantitative assessment of progress toward Sustainable Development Goals (SDGs) in China suggests that some SDGs underpinning goals for water pollution and biodiversity have not been met [2]. Specifically, tests on water quality improvement in lakes have had contradictory results [3,4], leading to confusion about water quality improvement in China.

Here, we synthesize water pollution governance and evaluate the effectiveness of pollution mitigation actions for rivers and lakes in China. We focus on the main stem of the Yangtze River and 25 associated lakes from the mid to lower reaches of the Yangtze River (MLRYR). Water quality in 2008–2018 is assessed using concentrations of chemical oxygen demand (COD), ammonium-nitrogen (NH_4_^+^-N) and total phosphorus (TP) for rivers, and COD, total nitrogen (TN), TP and chlorophyll *a* (Chl*a*) concentration for lakes. Lake trophic state is evaluated using a comprehensive index, and lake ecological condition is assessed with the Shannon-Weaver biodiversity index for benthic macroinvertebrates (Supplementary Materials).

We found that the water quality of rivers has improved across the country. There has been a significant increase in the percentage of grade I (highest water quality) to III cases and a decrease in grade V^+^ (poorest water quality) cases for river cross sections (Fig. S1) and river lengths (Fig. S2). Monthly monitoring of 14 cross sections along the Yangtze River main stem (Table S2) in 2007–2018 demonstrates that COD, NH_4_^+^-N and TP concentrations have generally declined (Fig. 1b). It is evident that China has made substantial progress towards combating water pollution of rivers.

In contrast, the water quality and ecological condition of lakes have not improved due to low hydraulic flushing rates. Evaluation of lake trophic conditions across the country in 2008–2018 showed an increase in the percentage of eutrophic lakes and a decrease in the percentage of oligotrophic lakes (Fig. S3), indicating that deterioration of lake water quality resulting from eutrophication has neither been halted nor reversed.

We further examined the water quality of 24 lakes from MLRYR (Table S3, Fig. 1c) between 2008 and 2018, and found no change in COD and TN (*p* > 0.05) in nearly half of the lakes (45.8% for COD and 54.2% for TN), and a significant increase (*p* < 0.05) in TP and Chl*a* in most lakes (50% for TP and 66.7% for Chl*a*) (Fig. 1c). Moreover, biodiversity of benthic fauna (Table S4) showed no change in an overwhelming majority of lakes (81.0%) (Fig. 1c) between 2008 and 2018. Monthly monitoring of the COD, TN, TP and Chl*a* of 11 representative large lakes from MLRYR in 2008–2018 (Fig. S4, Table S5) indicated that more than half had increased in COD (6 out of 11 lakes), TN (7 out of 11 lakes), TP (9 out of 11 lakes) and Chl*a* (8 out of 10 lakes) (Fig. S4, Table S5). The water quality and ecological state of most lakes seem unresponsive to pollution governance in 2008–2018.

**Figure 1.**
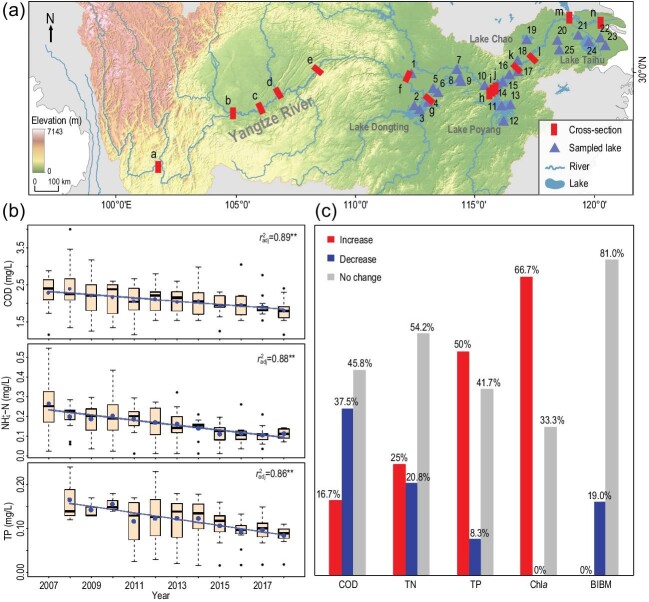
(a) Location of 14 cross sections from the Yangtze River main stem and 25 lakes from the mid to lower reaches of the Yangtze River. For names and locations of cross sections refer to Table S2. Lakes are numbered as follows: 1, Lake Changhu; 2, Lake Datong; 3, Lake Dongting; 4, Lake Yueyangnanhu; 5, Lake Honghu; 6, Lake Huanggai; 7, Lake Donghu; 8, Lake Liangzi; 9, Lake Cihu; 10, Lake Wushan; 11, Lake Poyang; 12, Lake Junshan; 13, Lake Zhuhu; 14, Lake Longgan; 15, Lake Huangda; 16, Lake Wuchang; 17, Lake Shengjin; 18, Lake Caizi; 19, Lake Chaohu; 20, Lake Shijiu; 21, Lake Gehu; 22, Lake Yangcheng; 23, Lake Dianshan; 24, Lake Taihu; 25, Lake Nanyi. For lake geographical attributes refer to Table S3. (b) Changes in water quality indices (COD, NH_4_^+^-N and TP) at 14 sites in the main stem of the Yangtze River over the period 2007–2018. Annual median value is indicated by a bar, and the box denotes the quartile ranges. The mean and standard deviation are shown by the blue dot and dashed line, respectively. (c) Comparison of changes in COD, TN, TP and Chl*a* concentrations for 24 lakes (except for Lake Taihu) and the biodiversity index for benthic macroinvertebrates (BIBM) for 21 lakes (see Table S4) between 2008 and 2018 in the middle and lower reaches of the Yangtze River, indicated as percentage of significant increase (*t*-test, *p* < 0.05), significant decrease (*t*-test, *p* < 0.05) or no change (*t*-test, *p* ≥ 0.05), and marked at the top of the columns.

Lake Taihu exemplifies such unresponsiveness to pollution governance. Monitoring of the water quality of rivers feeding the lake in 2008–2018 showed an increase in the number of grade I to III cases and a decrease in the number of grade V and V^+^ cases (Fig. S5). Water quality monitoring of the lake revealed decreased concentrations of TN, no trend of TP and increased Chl*a* (Fig. S6). The Shannon-Weaver biodiversity index of benthic macroinvertebrates showed a slight decline from 2013 to 2018 (Fig. S7). Responses of water quality and benthic biodiversity in Lake Taihu suggest that the lake restoration has been unsuccessful, although this lake has received the largest investment towards pollution governance in China.

Since the Lake Taihu drinking water crisis [5] in May 2007, a series of countermeasures aimed at lake water quality improvement have been implemented. These countermeasures include construction of wastewater collection pipeline networks and treatment plants, sediment removal and geoengineering, and water diversion from the Yangtze River to increase the flushing rate and water supply [6]. The effluent diversion mainly focused on control of point source pollution in urban areas. However, these efforts have not resulted in a significant decrease in external loading (Fig. S6), which is attributed to three factors. The first is low wastewater treatment standards and increasing water consumption linked to economic growth (Table S6), resulting in the contribution of point source pollution ranging from one-third to one-half of the external load. The second is the non-point source pollution in rural areas, which accounts for more than 50% of external loading. To date, only ca. 10% of non-point source pollution has been reduced through restoring wetlands. The third is the inter-basin water diversion from the Yangtze River, which increases the external loading as nitrogen and phosphorus concentrations of the Yangtze River are higher than Lake Taihu.

Internal loading from sediment is another key issue for shallow lakes. In Lake Taihu, ∼60%–70% of external phosphorus is retained at the lake bottom. This ‘legacy phosphorus’ is increasingly mobilized with the proliferation of cyanobacterial blooms [7]. The shallow depth (maximum <3 m) and frequent sediment resuspension mean that *in-situ* measures such as sediment capping or flocculation are largely ineffective. Furthermore, *ex-situ* treatments, such as sediment dredging, which had been conducted in a limited area (<100 km^2^), are hindered by inadequate storage capacity for dredged sediments and the secondary pollution risk.

In addition, climate warming has increased cyanobacterial blooms and extended the ‘window’ to almost year-round for blooms to form and persist. In turn, cyanobacterial bloom decay has led to anoxia and mobilization of nutrients from the sediments, promoting additional cyanobacterial blooms in a positive feedback loop [7].

A similar consequence of lake restoration is evident in Lake Chaohu [8], another large, shallow and eutrophic lake from MLRYR, suggesting that the dilemma of lake restoration in Lake Taihu is representative of a broader challenge in China. The reason for the lake restoration dilemma is the inadequate pollution control of both external and internal loading, which has its roots in the conflict of economic growth and pollution governance in developing areas.

To sustainably improve the water quality of eutrophic lakes in developing areas, pollution governance should be aligned with social and economic development. Considering Lake Taihu as an example, a water-food-energy-climate-economy nexus could systematically address the SDGs by including pollution governance within the watershed, in association with water quantity management (droughts, floods and inter-basin diversions), green farming and manufacturing (less point and non-point source pollution), sediment management, economic transformation, and adaption to climate change. However, tools addressing diverse sectors at larger, integrated scales are lacking, and need to be developed urgently. Long-term persistent improvement of the water quality and ecosystems of lakes requires nature-based solutions coupled with engineered systems to pursue sustainable development [9]. One positive sign is the recently initiated Yangtze River Delta regional integrated development strategy [10], which includes water pollution governance in the Lake Taihu basin.

## Supplementary Material

nwab207_Supplemental_File
