# Supplementary material for: Polluted lake restoration to promote sustainability in the Yangtze River Basin, China
Source: Natl Sci Rev. 2021 Nov 24;9(1):nwab207. doi: 10.1093/nsr/nwab207 (PMC8776540; doi:10.1093/nsr/nwab207)
Supplement: nwab207_Supplemental_File [file nwab207_supplemental_file.docx]

Supplementary Materials for

**Polluted Lake Restoration to Promote Sustainability in the Yangtze River Basin, China**

Boqiang Qin^a,b*^, Yunlin Zhang^a^, Jianming Deng^a^, Guangwei Zhu^a^, Jianguo Liu^c^, David P. Hamilton^d^, Hans W. Paerl^e,f^, Justin D. Brookes^g^, Tingfeng Wu^a^, Kai Peng^a^, Yizhou Yao^a^, Kan Ding^a^, Xiaoyan Ji^h^

*Corresponding author. Boqiang Qin, Email: qinbq@niglas.ac.cn

**This PDF file includes:**

**1. Data and Materials**

**2. Figs. S1 to S7**

**3. Tables S1 to S6**

**1. Data and Materials**

**1.1 Study area**

The Yangtze River (Changjiang River), the third largest river in the world and the largest river in China (length ~6300 km and catchment area ~18x10^6^ km^2^). The population and domestic gross production (GDP) of Yangtze River catchment account for 33.0% and 35.4% of whole China, respectively. The mid to lower reaches of Yangtze River (MLRYR) include most eutrophic lakes from China ([1](#_ENREF_1)). Number of lakes with area > 1 km^2^ in the MLRYR account for ca. 60% of freshwater lakes in China ([2](#_ENREF_2)). All lakes are shallow (Table S3) ([3](#_ENREF_3)). Lake Taihu is a typical large (area is 2336 km^2^), shallow (maximum depth is less than 3 m) and eutrophic lake located in the Yangtze River delta.

**1.2 Data acquisition**

Three types of data were used in this analysis. The first is governmental policies and laws, collected from publicly accessible sites (Table S1). We synthesized policies and laws directly linked to water pollution governance at national scale, or specifically for the Yangtze River basin or Lake Taihu.

The second is water quality and trophic state assessment data of rivers and lakes across the country. Nationwide water quality monitoring and evaluation were conducted independently by the Ministry of Ecology and Environment of China (MEE) with lake number varied from 23 in 2008 to 104 in 2018 (in 2012, the number of lakes increased sharply by adding many reservoirs from the middle and western China) ([4](#_ENREF_4)) and the Ministry of Water Resource of China (MWR) with lake number varied from 44 in 2008 to 156 in 2018 ([5](#_ENREF_5)). This included water quality assessment data of tributaries to Lake Taihu with the number of tributaries varied from 27-33 around the lake. These data were collected from publicly accessible sites.

The third is the observational data. We have collected monthly monitoring water quality of 14 cross-sections from the Yangtze River mainstem (Table S2) and 11 representative large lakes from the MLRYR (table S5). Water quality of the Yangtze River main stem was monitored by the China National Environmental Monitoring Centre (CNEMC), MEE, and Bureau of Hydrology, Yangtze River Water Resource Commission (YRWRC), MWR. Monthly water quality data of 11 lakes over the period 2008-2018 were collected from a variety of sources (Table S3). Lake Poyang was monitored by Poyang Lake Ecosystem Research Station, CAS; Lake Dongting was monitored by Dongting Lake Observation and Research Station of Wetland Ecosystem, CAS; Lake Gehu was collected from publication ([6](#_ENREF_6)) and CNEMC, MEE. Monitoring data of Lakes Honghu, Liangzi, Longgan, Nanyi, Dianshan, Yangchenghu were collected from CNEMC, MEE. In addition, water quality of 24 lakes (Table S3) and benthic macroinvertebrate fauna of 21 in 24 lakes (Table S4) were investigated in 2008 and 2018 by the Nanjing Institute of Geography & Limnology, Chinese Academy of Sciences, and each site was sampled during dry (November to March) and wet (June to September) seasons.

However, there are still some differences among the water sample pre-treatments. Pretreatment of water samples by CNEMC, MEE, included first settling for 30 minutes and then filtering through a 63 μm mesh before measurement, but by YRWRC, MWR, were settling only for 30 minutes before measurement, and measured directly by other institutions without settling and filtering. Water sampling and chemical analysis generally followed standard methods use in China ([7](#_ENREF_7)). COD was measured with addition of KMnO_4_ ([8](#_ENREF_8)). TN and TP concentrations were determined by spectrophotometry after digestion with alkaline potassium persulfate ([7](#_ENREF_7)). Chlorophyll a (Chla) concentration was measured using spectrophotometry at wavelengths of 665 and 750 nm, following extraction with hot 90% ethanol. For macroinvertebrate, samples were collected with three 0.025 m^2^ modified Peterson grabs and were sieved *in-situ* through 250-μm-aperture mesh. Residual material retained in the sieve was transported to the laboratory on the same day. In the laboratory, the samples were sorted on a white tray, and the specimens were preserved in 7% buffered formalin solution ([9](#_ENREF_9)). The specimens were identified to the species or genus level and counted.

Lake trophic state is evaluated with the comprehensive trophic index (trophic level index, TLI) which includes water quality indices Chla, TN, TP, Secchi depth (SD) and COD, and is obtained by averaging all above indices with weights ([10](#_ENREF_10)). Shannon-Weaver biodiversity index for benthic macroinvertebrates (BIBM) is used to indicate the lake ecological condition ([11](#_ENREF_11)), and the R package ‘vegan’ is used to calculate the Shannon-Weaver diversity index ([12](#_ENREF_12)).

**1.3 Statistical analysis**

Nonlinear fits using generalized additive models (GAMs) were developed with time-series data ([13](#_ENREF_13)). Trend significance was examined with Mann-Kendall tests (nonparametric) from the R package “Kendall”. Comparison of mean values of COD, TN, TP and Chl*a* concentrations between 2008 and 2018 in 24 lakes from MLRYR was carried out using *t*-tests. Benthic biodiversity of Lake Taihu was measured with the Shannon-Weaver diversity index.

**2. Figs. S1 to S7**


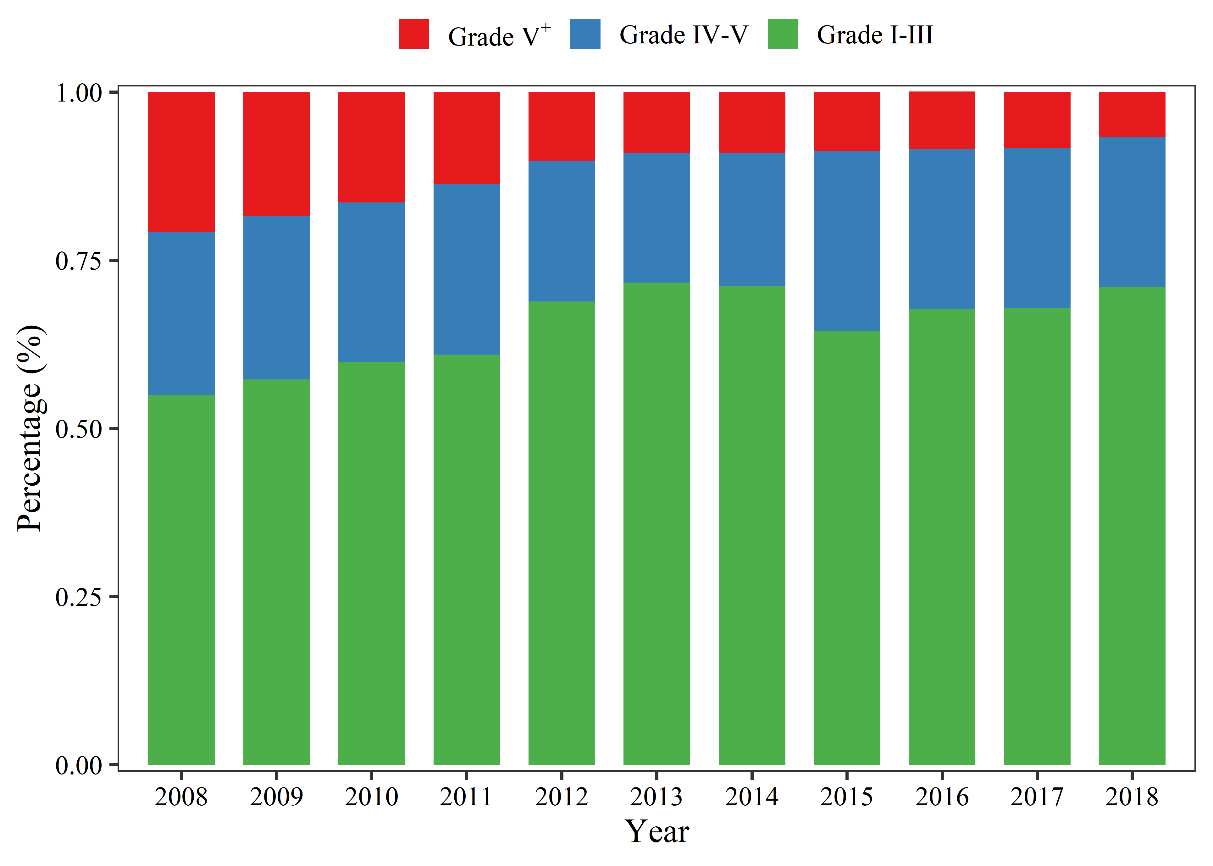


**Fig. S1**. Changes in percentage of water quality categorizations in 200 rivers during 2008–2018 over a variety of cross-sections from 409 sites in 2008 to 1935 sites in 2018. Data were collected from the Ministry of Ecology and Environment (MEE) ([4](#_ENREF_4)). According to China’s National Standard of Surface Water (GB3838-2002)([14](#_ENREF_14)), water quality is categorized into six classes from I to V^+^, ranked from best to worst.


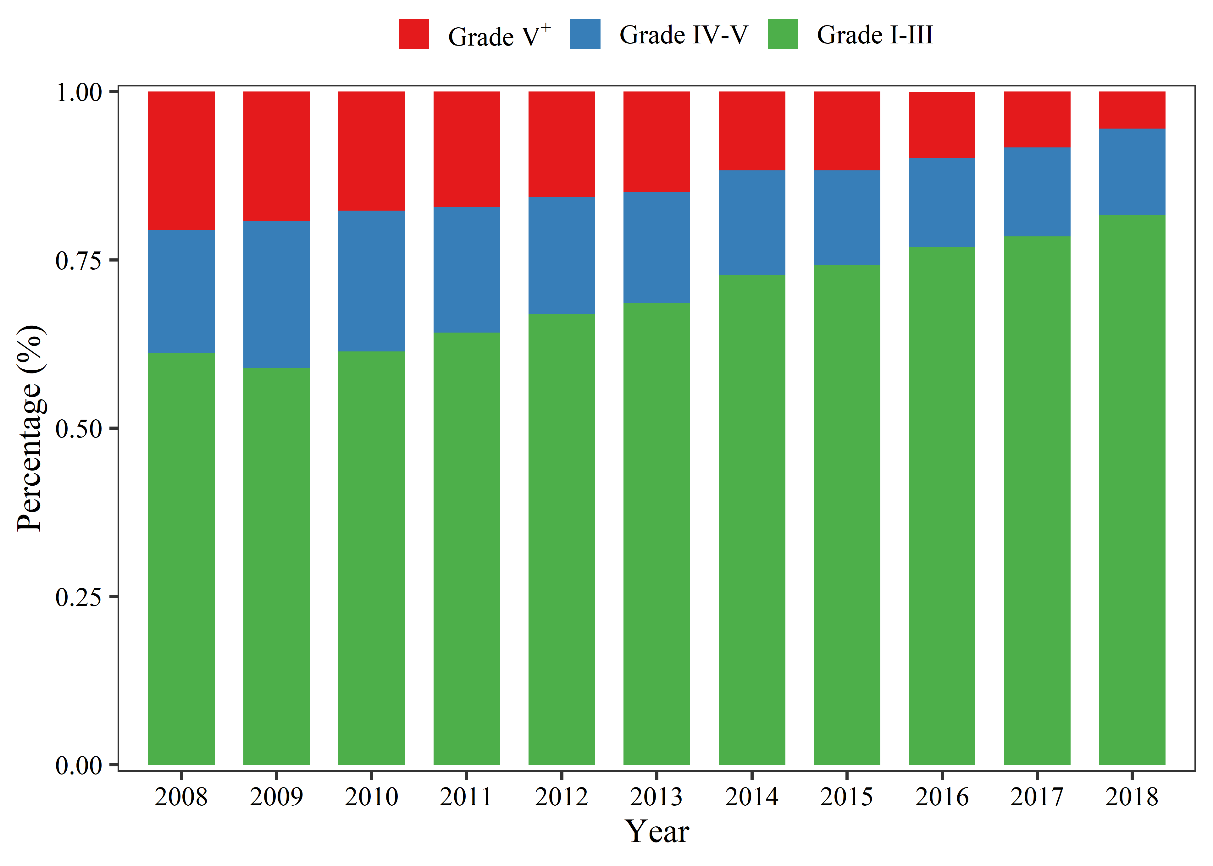


**Fig. S2**. Changes in percentage of water quality categorizations for water quality evaluations over cumulative river longitudinal lengths from 150,000 km in 2008 to 262,000 km in 2018. Data were collected from the Ministry of Water Resource of China ([5](#_ENREF_5)). Water quality is categorized into six grades from I to V^+^, ranked from best to worst ([14](#_ENREF_14)).


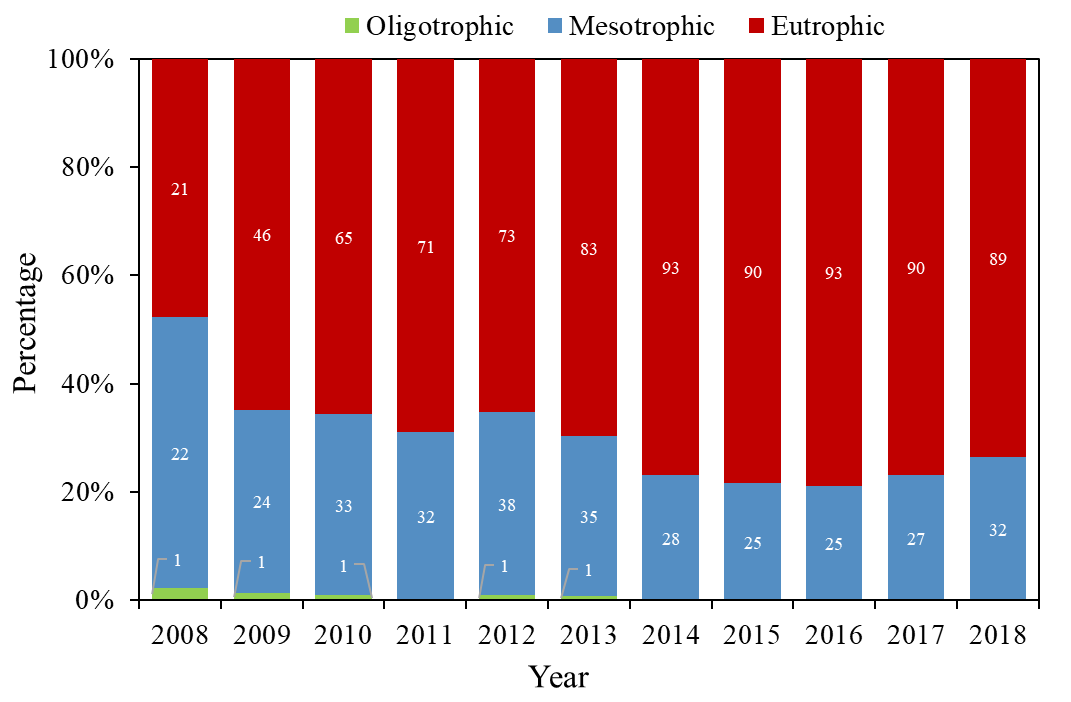


**Fig. S3**. Changes in percentage of lake trophic states for the oligotrophic, mesotrophic and eutrophic categorizations (total number of lakes varied from 44 in 2008 to 121 in 2018 and number of each categorization was marked in column). Data were collected from the Ministry of Water Resource of China ([5](#_ENREF_5)).


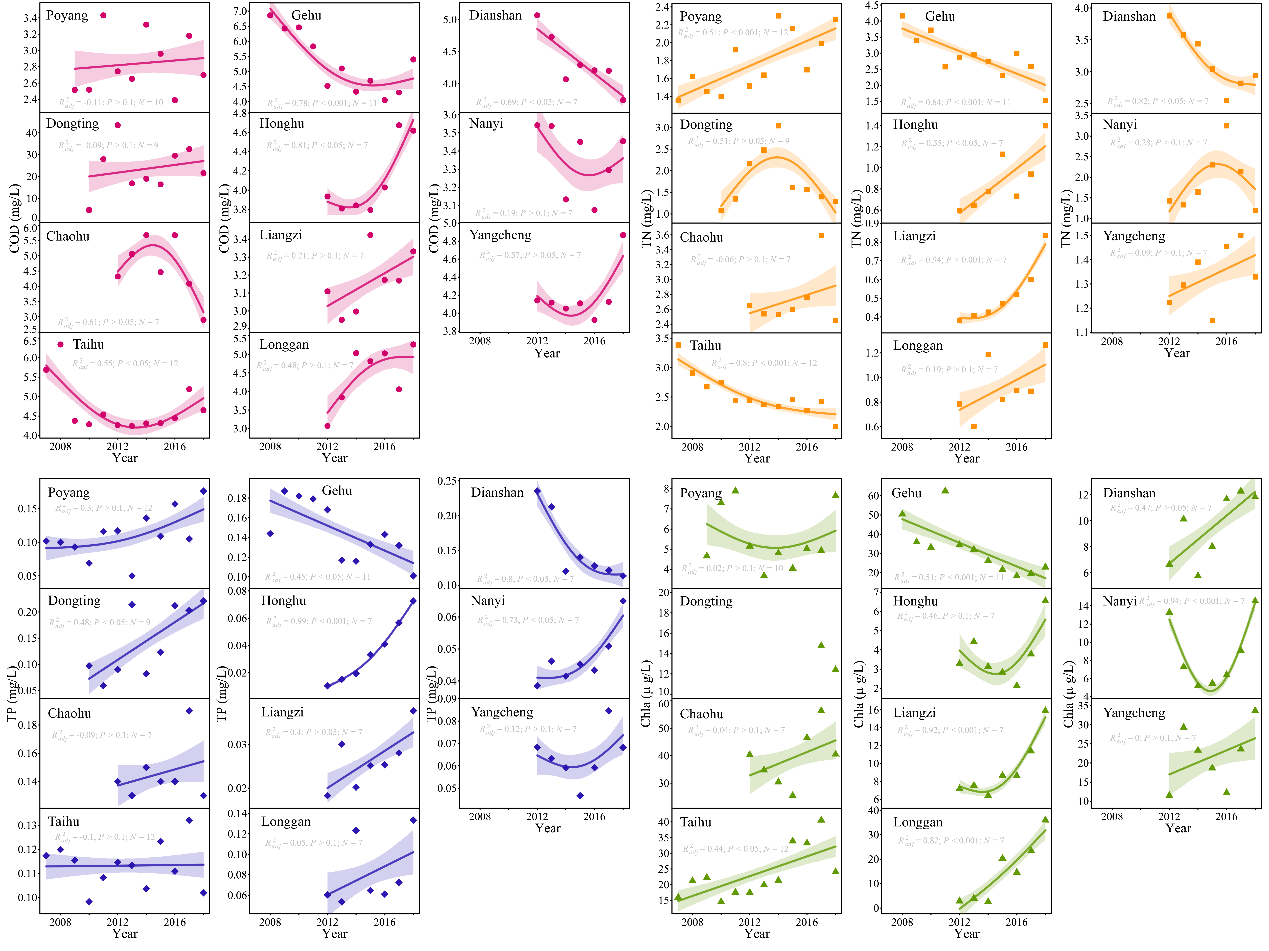


**Fig. S4.** COD, TN, TP and Chla concentrations of 11 lakes fluctuated over the period 2007–2018. Data sources: Lake Poyang (Poyang Lake Ecosystem Research Station, CAS), Lake Dongting (Dongting Lake Observation and Research Station of Wetland Ecosystem, CAS), Lake Gehu ([6](#_ENREF_6)). Lakes Honghu, Liangzi, Longgan, Nanyi, Dianshan, Yangchenghu (China National Environmental Monitoring Centre (CNEMC), Ministry of Ecology and Environment of China). Nonlinear fits using the generalized additive models (GAMs) from the “mgcv” package in R 3.6.1 ([15](#_ENREF_15)) were developed with time-series data according to ([13](#_ENREF_13)). The solid lines represent the long-term trends given by additive models (AMs), and the shaded area is the standard error of the estimate.


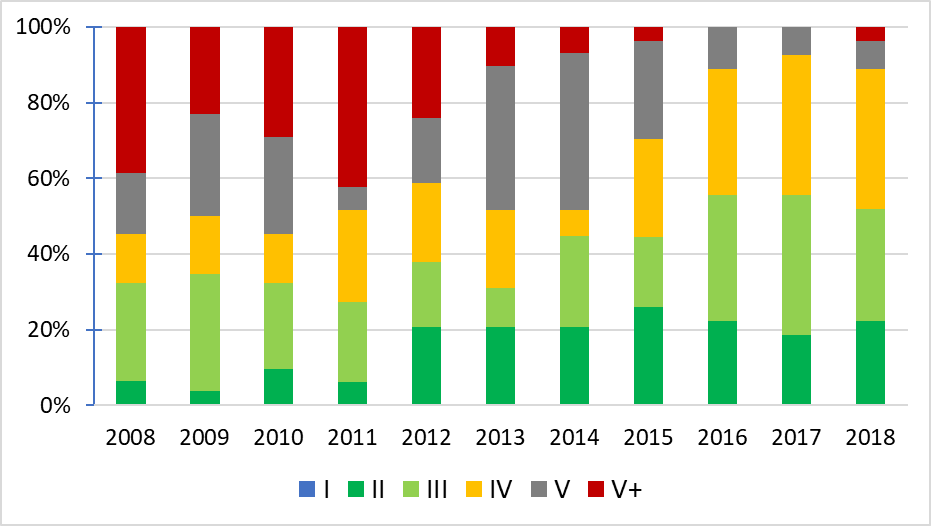


**Fig. S5.** Changes in percentage of water quality categorizations of rivers (ranging 27-33) around Lake Taihu during 2008–2018. Data were collected from the Taihu Basin Authority, Ministry of Water Resource (MWR) ([16](#_ENREF_16)). According to China’s National Standard of Surface Water Quality (GB3838-2002) ([14](#_ENREF_14)), water quality is categorized into six classes from I to V^+^, ranked from best to worst.


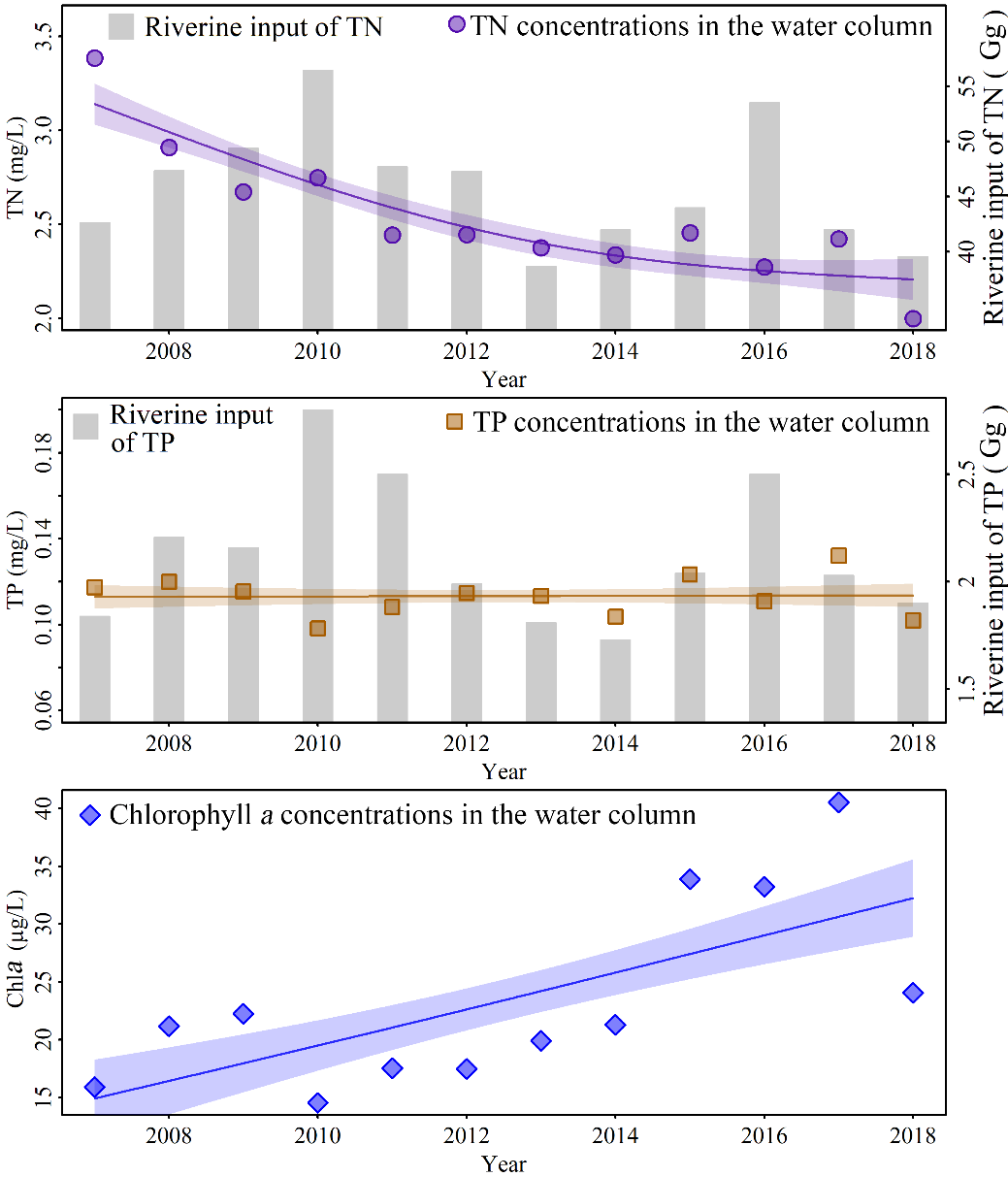


**Fig. S6.** Changes in annual concentrations of TN, TP and Chl*a* of Lake Taihu and riverine input of TN (Gg/yr) and TP (Gg/yr) during 2007-2018. Points are annual mean concentrations of TN, TP and Chl*a*, columns are the yearly input loads of TN and TP. Concentrations of TN, TP and Chl*a* were unresponsive to changes in external loading, indicating the influence from other factors, such as internal loadings. Riverine input loads were collected from Taihu Basin Authority, MWR ([16](#_ENREF_16)), and TN, TP and Chl*a* concentration data were collected from Taihu Laboratory for Lake Ecosystem Research (TLLER), Nanjing Institute of Geography & Limnology (NIGLAS), CAS.


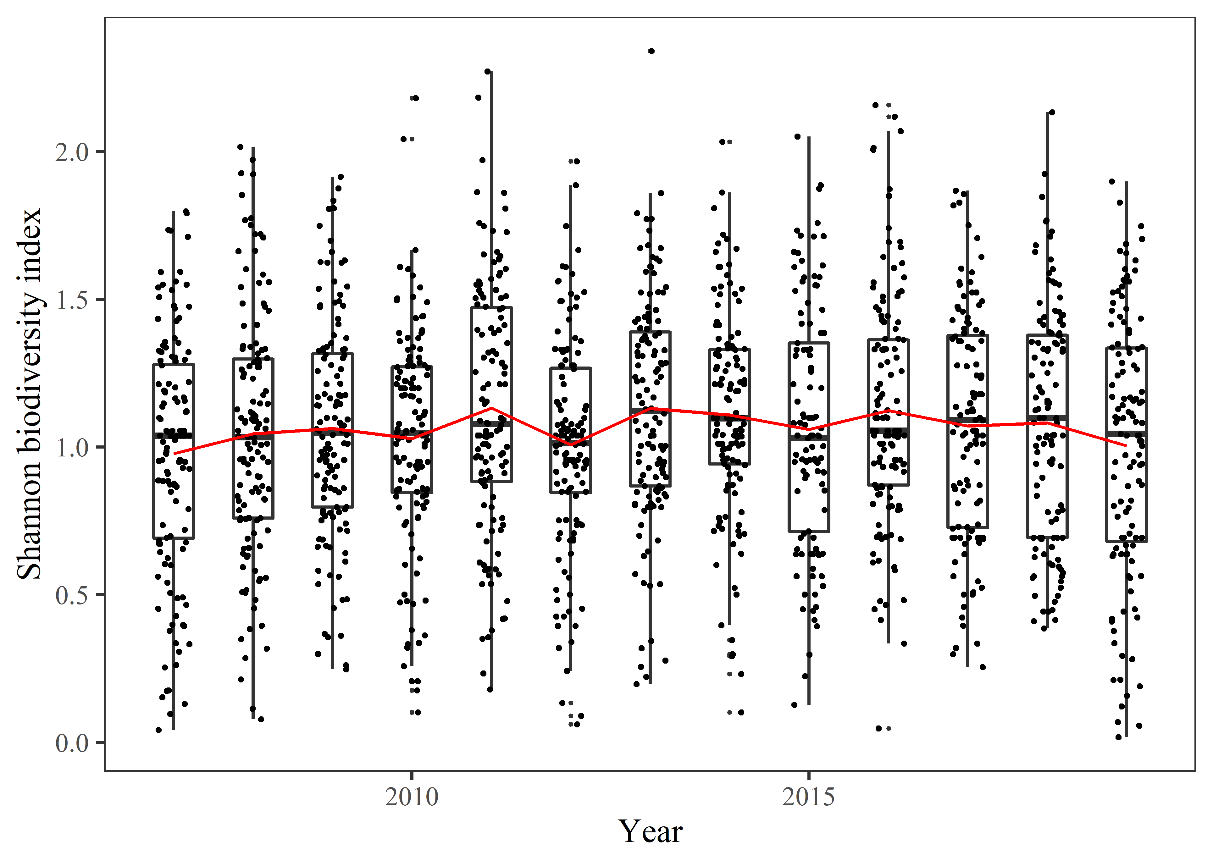


**Fig. S7.** Changes of Shannon-Weaver Biodiversity index of benthos over 32 sites over the entire area of Lake Taihu during 2007-2018. The plot box ranges one quartile and three quartile, and the median value is indicated by a bar inside the box. The mean and standard deviation are shown by the red line and black line. Data were collected from Taihu Laboratory for Lake Ecosystem Research (TLLER), Nanjing Institute of Geography & Limnology (NIGLAS), CAS.

**3. Tables S1 to S6**

| 1. **Title**: Law of the People's Republic of China on Prevention and Control of Water Pollution.   **Issued by administration**: Standing Committee of the National People's Congress.  **Time for targets or implementation**: Firstly issued in 2008, revised in 2017.  **URL**: <http://www.npc.gov.cn/npc/sjxflfg/201906/863e41b43f744efda56b14762e28dc6f.shtml>  **Description**: Prevent and control water pollution; protect water ecology; guarantee drinking water safety; maintain public health; and promote sustainable development by clarifying the responsibility of government, strengthening supervision and public participation, perfecting specific law and regulation, applying advanced treatment technology, building river chief system, and preparing appropriate development plan and financial support.   1. **Title**: The Water Pollution Prevention and Control Action Plan.   **Issued by administration**: State Council.  **Time for targets or implementation**: 2015.  **URL**: http://www.mee.gov.cn/zcwj/gwywj/201811/t20181129_676575.shtml  **Description**: Tackle water pollution via pollutant discharge control, industrial structure adjustment, protection and restoration, environmental science and technology development, market development, law enforcement, resource management, and public participation and social supervision.   1. **Title**: Regulations on Urban Drainage and Sewage Treatment.   **Issued by administration:** State Council.  **Time for targets or implementation:** 2013.  **URL**: http://www.gov.cn/zhengce/content/2013-10/18/content_5001.htm  **Description:** Strengthen the management of urban drainage and sewage treatment; prevent and control urban water pollution and waterlogging disasters; guarantee the safety of citizens' lives, property and public rights; and protect the environment by strengthening the leadership of government, improving treatment facilities, enacting rules to prevent pollution, developing financial methods, and enhancing supervision.   1. **Title**: Implementation Plan for Treatment of Urban Black and Odorous Water.   **Issued by administration:** Ministry of Housing and Urban Rural Development, Ministry of Ecology and Environment.  **Time for targets or implementation:** 2018.  **URL:** <http://www.mohurd.gov.cn/wjfb/201810/t20181015_237912.html>  **Description:** Improve water quality and citizens’ living standard via treatment, restoration, source reduction, and management reform.   1. **Title**: Integrated Wastewater Discharge Standard.   **Issued by administration:** Ministry of Ecology and Environment.  **Time for targets or implementation:** 1998  **URL**: <http://openstd.samr.gov.cn/bzgk/gb/newGbInfo?hcno=1BF9CDBD6FDE2AD5053C69A36318972C>  **Description:** Make up the allowable emission concentration of a series of significant pollutants to control water pollution; protect water quality of rivers, lakes, canals, channels, reservoirs, and oceans; protect human health; maintain ecosystem balance; and promote the development of economy.   1. **Title**: Action Outline of Conservation and Protection of Aquatic Biological Resources in China. **Issued by administration:** State Council.   **Time for targets or implementation:** 2006-2050.  **URL**: <http://www.gov.cn/zwgk/2006-02/27/content_212335.htm>  **Description:** Realize the sustainable development of fisheries and protect the diversity of aquatic organisms via scientific innovation, law and regulation, ecosystem restoration, and environmental protection.   1. **Title**: Water Pollution Prevention and Control in the "Three Rivers and Three Lakes".   **Issued by administration:** State Council & Ministry of Ecology and Environment.  **Time for targets or implementation:** 1996-present  **URL:** http://www.npc.gov.cn/wxzl/gongbao/2001-01/02/content_5003506.htm  **Description**: A series of plans that control water pollution and curb the water quality deterioration trend in Huaihe River, Haihe River, Liaohe River, Taihu Lake, Chaohu Lake, and Dianchi Lake basin by adjusting local industrial structure, improving urban sewage treatment plants, limiting agricultural non-point source pollution from fertilizers and pesticides, regulating livestock and poultry breeding, and applying comprehensive treatment measures.   1. **Title:** Water Pollution Prevention and Control Plan for the Middle and Lower Reaches of Yangtze River (2011-2015).   **Issued by administration:** Ministry of Ecology and Environment, National Development and Reform Commission, Ministry of Finance, Ministry of Housing and Urban Rural Development, Ministry of Water Resources.  **Time for targets or implementation:** 2011-2015.  **URL:** <http://www.mee.gov.cn/gkml/hbb/bwj/201109/t20110920_217490.htm>  **Description:** Strengthen the pollution control measures; promote the transformation of economic development; improve water environment quality and ecological safety; and protect the health of people by increasing the environment capacity and reducing total sewage discharge; clarifying the responsibility of local government; strengthening environmental protection programs and financial support.   1. **Title:** Ecological and Environmental Protection Plan for Yangtze River Economic Belt.   **Issued by administration:** Ministry of Ecology and Environment, National Development and Reform Commission, Ministry of Water Resources.  **Time for targets or implementation**: 2017-2030.  **URL:** <http://www.mee.gov.cn/gkml/hbb/bwj/201707/t20170718_418053.htm>  **Description:** Improve the environmental quality and ecological service and function of Yangtze River Basin by limiting resource utilization; setting up specific ecological protection area; perfecting environmental quality control standards, and controlling total pollutant discharge.   1. **Title:** Action Plan for the Battle of Protection and Restoration of Yangtze River.   **Issued by administration:** Ministry of Ecology and Environment, National Development and Reform Commission.  **Time for targets or implementation**: 2019-2020.  **URL**: <http://www.mee.gov.cn/xxgk2018/xxgk/xxgk03/201901/t20190125_690887.html>  **Description:** Improve the ecological function of wetland; guarantee the ecological water demand; and control the risk of ecological damage via water treatment, ecological restoration, water resource protection, pollution source limit, and comprehensive environmental measures.   1. **Title:** Opinions on Strengthening the Environmental Protection of Key Lakes.   **Issued by administration:** Ministry of Ecology and Environment, National Development and Reform Commission, the Ministry of Finance, Ministry of Housing and Urban Rural Development, Ministry of Water Resources.  **Time for targets or implementation**: 2008-2030.  **URL:** <http://www.gov.cn/zwgk/2008-01/22/content_864884.htm>  **Description:** Control the increasing eutrophication trend and gradually restore the natural features of significant lakes (Taihu Lake, Chaohu Lake, etc.) via industrial pollution control, agricultural non-point source pollution control, environmental management measures, and ecological restoration projects**.**   1. **Title:** Opinions on Comprehensively Promoting the River Chief System **^1^**   **Issued by administration:** State Council.  **Time for targets or implementation**: 2016.  **URL:** <http://www.gov.cn/xinwen/2016-12/11/content_5146628.htm>  **Description:** Every single river should have a government leader as the river chief to take responsibility of management and protection. The policy clarified the responsibilities of river management across administrative regions to promote comprehensive programs for solving critical environmental problems.   1. **Title:** Guidelines on the Implementation of Lake Chief System in Lake **^1^**   **Issued by administration:** State Council.  **Time for targets or implementation**: 2018.  **URL:** <http://www.gov.cn/zhengce/2018-01/04/content_5253253.htm>  **Description:** Implementing lake chief policy to clarify the management responsibility of local government, strengthen lake protection, improve lake ecological functions, protect wildlife and people’s health, and realize lake sustainable use.   1. **Title:** Guidelines on Accelerating Agricultural Non-Point Source Pollution Control in the Yangtze River Economic Belt.   **Issued by administration:** Ministry of Ecology and Environment, National Development and Reform Commission, Ministry of Agriculture and Rural Affairs, Ministry of Housing and Urban Rural Development, Ministry of Water Resources.  **Time for targets or implementation**: 2018.  **URL:** <http://www.gov.cn/xinwen/2018-11/01/content_5336376.htm>  **Description:** Accelerate the control of rural non-point source pollution; continuously improve the water quality; restore the ecological system; and promote the green production in rural areas along the Yangtze River economic belt via protection, financial support, industrial reform, and management improvement.   1. **Title:** Notice of State Council on Work Related to the Prohibition of Fishing in the Yangtze River Basin.   **Issued by administration:** State Council.  **Time for targets or implementation**: 2020.  **URL:** <http://www.gov.cn/zhengce/content/2020-07/08/content_5525124.htm>  **Description:** Promote the protection of aquatic bio-resource and ecosystem restoration of the Yangtze River Basin by implementing 10-year fishing ban in key waters; establishing compensation fund, and strengthening the resettlement and social assistance programs to fishermen. |
| --- |

^1^ The main leaders of the government act as "river leaders", who are responsible for organizing and leading the management and protection of corresponding rivers and lakes.

Table S1.

Laws, regulations and policies of water pollution governance issued by a variety of administrative departments at nationwide, Yangtze Basin or Lake Taihu basin.

| Letter | Name of cross-section | Longitude | Latitude | Situation of river | *ρ* (*k*) of COD | *ρ* (*p*) of COD | *ρ* (*k*) of NH_4_^+^-N | *ρ* (*p*) of NH_4_^+^-N | *ρ* (*k*) of TP | *ρ* (*p*) of TP |
| --- | --- | --- | --- | --- | --- | --- | --- | --- | --- | --- |
| a | Longdong**^1^** | 101.669 | 26.57613 | Upstream | +0.015 | 0.300 | +0.002 | 0.464 | -0.001 | 0.057 |
| b | Guagongshan^1^ | 104.689 | 28.77382 | Upstream | -0.165 | 0.000 | +0.003 | 0.624 | -0.014 | 0.000 |
| c | Zhutuo^2^ | 105.8626 | 29.01755 | Upstream | -0.138 | 0.010 |  |  | -0.012 | 0.002 |
| d | Cuntan^1^ | 106.6024 | 29.61981 | Upstream | -0.049 | 0.123 | -0.019 | 0.004 | -0.007 | 0.098 |
| e | Shaiwangba^1^ | 108.2842 | 30.53384 | Upstream | -0.007 | 0.514 | -0.002 | 0.284 | -0.013 | 0.002 |
| f | Jingjiangku^1^ | 112.2385 | 30.31492 | Midstream | -0.038 | 0.054 | -0.030 | 0.002 | -0.007 | 0.097 |
| g | Chenglingji^1^ | 113.152 | 29.44082 | Midstream | -0.023 | 0.214 | -0.021 | 0.019 | -0.006 | 0.060 |
| h | Jiujiang^1^Yaogang | 115.9746 | 29.73442 | Midstream | -0.077 | 0.016 | -0.014 | 0.016 | +0.003 | 0.140 |
| i | Wuqimatou^2^ | 115.9906 | 29.73922 | Midstream | +0.018 | 0.123 |  |  | -0.003 | 0.233 |
| j | Hukou^1^ | 116.0578 | 29.76999 | Midstream | -0.031 | 0.129 | -0.015 | 0.002 | +0.006 | 0.166 |
| k | Wanhekou^1^ | 117.0151 | 30.49812 | downstream | -0.071 | 0.000 | -0.013 | 0.006 | -0.006 | 0.011 |
| l | Datong^2^ | 117.7371 | 30.83742 | downstream | -0.016 | 0.596 |  |  | -0.004 | 0.021 |
| m | Jiaoshanwei^1^ | 119.4796 | 32.27010 | downstream | -0.018 | 0.533 | -0.012 | 0.000 | +0.003 | 0.355 |
| n | Nantong Yaogang^1^ | 120.8372 | 31.98098 | downstream | -0.020 | 0.263 | -0.018 | 0.026 | +0.002 | 0.028 |

^1^ Data is from China National Environmental Monitoring Centre (CNEMC), Ministry of Ecology and Environment of China

^2^ Data is from Bureau of Hydrology, Yangtze River Water Resources Commission (YRWRC), Ministry of Water Resource of China

Table S2.

Statistical significance tests (Mann-Kendall test) of temporal regression of COD, NH_4_^+^-N, and TP over 2007–2018, based on water quality monitoring of 14 cross-sections along the Yangtze River. The temporal regression slope (*ρ*(*k*) indicates the temporal change trend (increase with positive value and decrease with negative value). Significant test of temporal changes (*ρ*(*p*)) with significant level higher than 5% is marked with yellow.

| Number | Lake name | Province | Longitude (° E) | Latitude （° N） | Area  km^2^ | Average depth (m) | Data availability |
| --- | --- | --- | --- | --- | --- | --- | --- |
| 1 | Changhu | Hubei | 112.40 | 30.44 | 143.8 | 1.90 | 2008^1^, 2018^1^ |
| 2 | Datonghu | Hunan | 112.51 | 29.21 | 83.2 | 2.89 | 2008^1^, 2018^1^ |
| 3 | Dongting | Hunan | 112.74 | 29.07 | 2614.4 | 6.39 | 2008^1^, 2018^1^, 2007-2018^3, 3^ |
| 4 | Yueyangnanhu | Hunan | 113.12 | 29.34 | 16.6 | 1.70 | 2008^1^, 2018^1^ |
| 5 | Honghu | Hubei | 113.34 | 29.86 | 340.1 | 1.91 | 2008^1^, 2018^1^, 2012-2018^2^ |
| 6 | Huanggai | Hunan | 113.55 | 29.70 | 77.2 | 4.20 | 2008^1^, 2018^1^ |
| 7 | Donghu | Hubei | 114.40 | 30.56 | 34.4 | 2.80 | 2008^1^, 2018^1^ |
| 8 | Liangzi | Hubei | 114.51 | 30.23 | 351.8 | 4.16 | 2008^1^, 2018^1^, 2012-2018^2^ |
| 9 | Cihu | Hubei | 114.57 | 30.10 | 9.2 | 1.75 | 2008^1^, 2018^1^ |
| 10 | Wushan | Hubei | 115.59 | 29.91 | 15.8 | 3.10 | 2008^1^, 2018^1^ |
| 11 | Poyang | Jiangxi | 116.28 | 29.11 | 3207.0 | 5.10 | 2008^1^, 2018^1^, 2007-2018^2^ |
| 12 | Junshan | Jiangxi | 116.34 | 28.53 | 177.3 | 4.00 | 2008^1^, 2018^1^ |
| 13 | Zhuhu | Jiangxi | 116.67 | 29.14 | 67.0 | 5.72 | 2008^1^, 2018^1^ |
| 14 | Longgan | Anhui | 116.15 | 29.95 | 280.5 | 3.78 | 2008^1^, 2018^1^, 2012-2018^2^ |
| 15 | Huangda | Anhui | 116.38 | 30.02 | 288.6 | 3.94 | 2008^1^, 2018^1^ |
| 16 | Wuchang | Anhui | 116.69 | 30.28 | 112.0 | 3.43 | 2008^1^, 2018^1^ |
| 17 | Shengjin | Anhui | 117.07 | 30.38 | 96.1 | 1.26 | 2008^1^, 2018^1^ |
| 18 | Caizi | Anhui | 117.07 | 30.80 | 171.6 | 1.67 | 2008^1^, 2018^1^ |
| 19 | Chaohu | Anhui | 117.53 | 31.57 | 789.1 | 2.69 | 2008^1^, 2018^1^, 2012-2018^1^ |
| 20 | Shijiu | Jiangsu | 118.88 | 31.47 | 214.4 | 2.42 | 2008^1^, 2018^1^ |
| 21 | Gehu | Jiangsu | 119.81 | 31.60 | 139.0 | 1.19 | 2008^1^, 2018^1^, 2008-2018^4^ |
| 22 | Yangchenghu | Jiangsu | 120.77 | 31.43 | 118.1 | 1.40 | 2008^1^, 2018^1^, 2012-2018^2^ |
| 23 | Dianshan | Shanghai | 120.96 | 31.12 | 59.2 | 2.50 | 2008^1^, 2018^1^, 2012-2018^2^ |
| 24 | Taihu | Jiangsu | 120.25 | 31.32 | 2336.0 | 1.9 | 2007-2018^1^ |
| 25 | Nanyi | Anhui | 118.93 | 31.10 | 148.4 | 2.25 | 2012-2018^2^ |

^1^ Data were collected from the Nanjing Institute of Geography & Limnology (NIGLAS), Chinese Academy of Science (CAS)

^2^ Data were collected from the China National Environmental Monitoring Centre (CNEMC), Ministry of Ecology and Environment of China

^3^ Dongting Lake Observation and Research Station of Wetland Ecosystem, CAS.

^4^ Xu X, et al. (2020) Regime shifts in shallow lakes observed by remote sensing and the implications for management. Ecol. Indic. 113:106285.

Table S3.

List of names, locations and data availability of 25 lakes distributed in the middle and lower reaches of the Yangtze River, in which 24 lakes have monitoring data between 2008 and 2018, 11 lakes have consecutive monitoring data during 2008-2018.

| **Lake name** | **Province** | **Longitude** (° E) | **Latitude**  (° N) | **ΔD (Diversity difference between 2018 and 2008)** | **ρ (*p*) of ΔD** |
| --- | --- | --- | --- | --- | --- |
| Caizi | Anhui | 117.07 | 30.8 | -0.518 | 0.049 |
| Chaohu | Anhui | 117.53 | 31.57 | -0.758 | 0.000 |
| Datonghu | Hubei | 114.57 | 30.1 | -0.023 | 0.865 |
| Dianshan | Shanghai | 120.96 | 31.12 | -0.404 | 0.331 |
| Donghu | Hubei | 114.4 | 30.56 | -0.036 | 0.876 |
| Dongting | Hunan | 112.74 | 29.07 | -0.251 | 0.293 |
| Gehu | Jiangsu | 119.81 | 31.6 | -0.401 | 0.053 |
| Honghu | Hubei | 113.34 | 29.86 | +0.075 | 0.694 |
| Huangda | Anhui | 116.38 | 30.02 | -0.662 | 0.055 |
| Huanggai | Hunan | 113.55 | 29.7 | -0.311 | 0.051 |
| Junshan | Jiangxi | 116.34 | 28.53 | -0.276 | 0.228 |
| Liangzi | Hubei | 114.51 | 30.23 | +0.378 | 0.063 |
| Longgan | Anhui | 116.15 | 29.95 | -0.283 | 0.064 |
| Poyang | Jiangxi | 116.28 | 29.11 | -1.133 | 0.001 |
| Shengjin | Anhui | 117.07 | 30.38 | -0.354 | 0.032 |
| Wuchang | Anhui | 116.69 | 30.28 | -0.327 | 0.245 |
| Wushan | Hubei | 115.59 | 29.91 | +0.193 | 0.221 |
| Yangchenghu | Jiangsu | 120.77 | 31.43 | -0.042 | 0.820 |
| Yueyangnanhu | Hunan | 113.12 | 29.34 | +0.120 | 0.631 |
| Changhu | Hubei | 112.4 | 30.44 | +0.088 | 0.613 |
| Zhuhu | Jiangxi | 116.67 | 29.14 | -0.155 | 0.452 |

Table S4.

Difference of benthic biodiversity index between 2008 and 2018 (ΔD, minus (-) means biodiversity index decrease and positive (+) means biodiversity index increase) in 21 lakes from the middle and lower reaches of the Yangtze River. Significant change in ΔD (***ρ (p)***) with significant level higher than 5% is marked with yellow.

| Lake name | **K-COD**  *ρ*(*k)* | **P-COD**  *ρ*(*p)* | **K-TN**  *ρ*(*k)* | ***P*-TN**  *ρ*(*p)* | **K-TP**  *ρ*(*k)* | ***P*-TP**  *ρ*(*p)* | **K-Chl*a***  *ρ*(*k)* | ***P-*Chl*a***  *ρ*(*p)* |
| --- | --- | --- | --- | --- | --- | --- | --- | --- |
| Poyang | +0.015 | 0.073 | +0.070 | 0.005 | +0.006 | 0.049 | -0.037 | 0.839 |
| Dongting | +0.884 | 0.578 | -0.018 | 0.846 | +0.018 | 0.025 | / | / |
| Taihu | -0.076 | 0.186 | -0.085 | 0.000 | +0.000 | 0.598 | +1.577 | 0.011 |
| Chaohu | -0.222 | 0.266 | +0.062 | 0.454 | +0.003 | 0.516 | +2.132 | 0.314 |
| Honghu | +0.142 | 0.030 | +0.106 | 0.035 | +0.010 | 0.000 | +0.268 | 0.369 |
| Liangzi | +0.046 | 0.168 | +0.066 | 0.006 | +0.002 | 0.075 | +1.287 | 0.016 |
| Longgan | +0.250 | 0.099 | +0.061 | 0.179 | +0.007 | 0.301 | +5.358 | 0.004 |
| Dianshan | -0.175 | 0.012 | -0.186 | 0.013 | -0.019 | 0.018 | +0.921 | 0.054 |
| Nanyi | -0.029 | 0.476 | +0.090 | 0.562 | +0.003 | 0.031 | +0.307 | 0.705 |
| Yangchenghu | +0.074 | 0.233 | +0.028 | 0.266 | +0.002 | 0.544 | +1.574 | 0.359 |
| Gehu | -0.231 | 0.005 | -0.176 | 0.002 | -0.006 | 0.014 | -3.068 | 0.008 |

Table S5.

Statistical significance tests of water quality trends during 2007-2018 in 11 lakes from MLRYR and EC. The temporal regression slope (*ρ*(*k*) indicates the temporal change trend (increase with positive value and decrease with negative value). Significant test of temporal changes (*ρ*(*p*)) with significant level higher than 5% is marked with yellow.

|  | Wuxi | | | | Changzhou | | | | Huzhou | | | |
| --- | --- | --- | --- | --- | --- | --- | --- | --- | --- | --- | --- | --- |
| Year | GDP （billion yuan) | Population (x10^6^) | WC (x10^6^ t) | PCDI (CNY) | GDP （x10^9^yuan) | Population (x10^6^) | WC (x10^6^t) | PCDI (CNY) | GDP （x10^9^yuan) | Population (x10^6^) | WC (x10^6^t) | PCDI (CNY) |
| 2007 | 388.0 | 357.38 | 289.36 | 20898 | 191.4 | 257.8 | 257.7 | 19089 | 88.3 | 461.74 | 83.5 | 19663 |
| 2008 | 446.1 | 358.74 | 300.63 | 23605 | 226.6 | 258.5 | 253.2 | 21592 | 102.3 | 464.2 | 79.3 | 21604 |
| 2009 | 499.2 | 359.82 | 336.35 | 25027 | 252.0 | 259.17 | 253.9 | 23751 | 110.1 | 465.65 | 91.0 | 23280 |
| 2010 | 579.3 | 360.8 | 399.77 | 27750 | 304.5 | 259.98 | 262.4 | 26269 | 130.2 | 466.56 | 93.7 | 25668 |
| 2011 | 688.0 | 362.86 | 373.03 | 31638 | 358.1 | 261.05 | 258.9 | 29829 | 152.0 | 467.96 | 95.3 | 29367 |
| 2012 | 756.8 | 364.77 | 394.72 | 35663 | 397.0 | 261.38 | 239.0 | 33587 | 166.4 | 470.07 | 96.1 | 32987 |
| 2013 | 777.0 | 365.91 | 371.61 | 38999 | 445.0 | 262.49 | 228.1 | 36946 | 181.3 | 472.23 | 96.1 | 36220 |
| 2014 | 820.5 | 368.64 | 349.51 | 36471 | 490.2 | 263.78 | 226.9 | 39483 | 195.6 | 477.14 | 108.0 | 38959 |
| 2015 | 851.8 | 370.85 | 363.05 | 39461 | 527.3 | 263.71 | 263.4 | 42710 | 208.4 | 480.9 | 142.0 | 42238 |
| 2016 | 915.7 | 374.9 | 375.26 | 42757 | 570.0 | 264.84 | 279.0 | 46058 | 224.3 | 486.2 | 116.8 | 45794 |
| 2017 | 1051.2 | 378.84 | 353.23 | 46453 | 662.3 | 266.14 | 282.9 | 49955 | 247.6 | 493.05 | 124.1 | 49934 |
| 2018 | 1143.9 | 382.2 | 386.81 | 50373 | 705.0 | 267.06 | 303.7 | 54000 | 271.9 | 497.21 | 137.4 | 54393 |

**Table S6.**

Increase of gross domestic production (GDP), population, water consumption (WC) and per capita disposable income (PCDI) of urban resident of three cities from upstream in the Lake Taihu basin

**Reference**

1. Guan, Q, Feng, L, Hou, X*, et al.* Eutrophication changes in fifty large lakes on the Yangtze Plain of China derived from MERIS and OLCI observations. *Remote Sens Environ*. 2020; **246**: 111890.

2. Wang, SM, Dou, HS. *Annals of Lakes in China*. Beijing: Science Press; 1998.

3. Huang, J, Xu, Q, Xi, B*, et al.* Effects of lake-basin morphological and hydrological characteristics on the eutrophication of shallow lakes in eastern China. *J Great Lakes Res*. 2014; **40**(3): 666-74.

4. Ministry of Ecology and Environment of China (MEE). *Report on the State of the Environment in China*. <http://english.mee.gov.cn/Resources/Reports/soe/>, Beijing: 2008-2018 (DHHS publication no.: Report Number)| (GPO o. Document Number)|.

5. Ministry of Water Resource of China (MWR). *Water Resources Bulletin of China*. <http://mwr.gov.cn/sj/tjgb/szygb/>, Beijing: 2008-2018 (DHHS publication no.: Report Number)| (GPO o. Document Number)|.

6. Xu, X, Zhang, Y, Chen, Q*, et al.* Regime shifts in shallow lakes observed by remote sensing and the implications for management. *Ecol Indic*. 2020; **113**: 106285.

7. Jin, XC, Tu, QY. *The standard methods for observation and analysis of lake eutrophication*. Beijing, China: China Environmental Science Press; 1990.

8. State Environmental Protection Administration (SEPA). *Water Quality: Determination of Permanganate Index (GB 11892-89) (in Chinese)*. Beijing: 1989 (DHHS publication no.: Report Number)| (GPO o. Document Number)|.

9. Cai, Y, Xu, H, Vilmi, A*, et al.* Relative roles of spatial processes, natural factors and anthropogenic stressors in structuring a lake macroinvertebrate metacommunity. *Sci Total Environ*. 2017; **601-602**: 1702-11.

10. Wang, MC, Liu, X.Q., Zhang,J.H. Evaluate method and classification standard on lake eutrophication. *Environmental Monitoring in China*. 2002; **18**(5): 47-9.

11. Poikane, S, Johnson, RK, Sandin, L*, et al.* Benthic macroinvertebrates in lake ecological assessment: A review of methods, intercalibration and practical recommendations. *Sci Total Environ*. 2016; **543**: 123-34.

12. Oksanen, J, Blanchet, F.G., Kindt, R., Legendre, P., Minchin, P.R., O’Hara, R.B., Simpson, G.L., Solymos, P., Stevens, M.H.H. and Wagner, H. *Vegan: Community ecology package, v. 2.4-5.* 2017 (DHHS publication no.: Report Number)| (GPO o. Document Number)|.

13. Harding, LW, Gallegos, CL, Perry, ES*, et al.* Long-term trends of nutrients and phytoplankton in Chesapeake Bay. *Estuaries and Coasts*. 2016; **39**(3): 664-81.

14. Ministry of Ecology and Environment of China (MEE). *Environmental Quality Standards for Surface Water (GB 3838-2002)*. <http://www.mee.gov.cn/ywgz/fgbz/bz/bzwb/shjbh/shjzlbz/200206/t20020601_66497.shtml>, Beijing: 2002 (DHHS publication no.: Report Number)| (GPO o. Document Number)|.

15. R Core Team. R: A language and environment for statistical computing. 2019.

16. Taihu Basin Authority, MoWR. *The Health Status Report of Lake Taihu*. <http://www.tba.gov.cn/slbthlyglj/thjkzkbg/content/slth1_09f7d6b21629439f9891c7fd70ad49d8.html:> 2008-2018 (DHHS publication no.: Report Number)| (GPO o. Document Number)|.
